# Effect of discriminate and indiscriminate use of oxytetracycline on residual status in broiler soft tissues

**DOI:** 10.14202/vetworld.2020.61-67

**Published:** 2020-01-10

**Authors:** Most. Rifat Ara Ferdous, Md. Raju Ahmed, Sayekul Hasan Khan, Mufsana Akter Mukta, Tasnia Tabassum Anika, Md. Tarek Hossain, Md. Zahorul Islam, Kazi Rafiq

**Affiliations:** Department of Pharmacology, Faculty of Veterinary Science, Bangladesh Agricultural University, Mymensingh - 2202, Bangladesh

**Keywords:** antibiotic residue, broiler edible tissues, oxytetracycline, thermal effect, thin-layer chromatography

## Abstract

**Aim:**

This study aimed to evaluate the effects of discriminate and indiscriminate use of oxytetracycline on hematological parameters, residual status in soft tissue of broiler and of thermal effect on oxytetracycline residual status.

**Materials and Methods:**

Eighteen, day-old male broiler chickens were purchased and were divided into three different groups (control group, discriminate group, and indiscriminate group). The control group received no antibiotics. The discriminate group received oxytetracycline 1 g/L drinking water for 5 consecutive days, and 10 days’ withdrawal period was maintained before sacrifice. The indiscriminate group received oxytetracycline 1 g/L drinking water till the sacrificed day. Blood samples were collected before sacrificing for hematological analysis. After sacrificing liver, kidney, spleen, and muscle samples were collected for analysis of oxytetracycline residues in raw soft tissues. Since meat is used to cook by traditional method in Bangladesh before consumption that is why positive meat samples were cooked by traditional cooking method to evaluate the thermal effect on oxytetracycline residual status as well. Thin-layer chromatography (TLC) was done for screening of oxytetracycline residues in soft tissues.

**Results:**

Mean differences of total erythrocyte count (million/mm^3^), hemoglobin estimation (gm%), and packed cell volume (%) estimation were not statistically significant among the groups. TLC analysis of raw samples showed 100% positive results of all samples collected from the indiscriminate group. In contrast, samples collected from the discriminate group were negative for oxytetracycline residues. In the control group, all samples were negative for oxytetracycline residue. There was a significant (p<0.05) relationship of oxytetracycline residues among three different groups for liver, kidney, spleen, and muscle samples. Positive liver and muscle samples from the indiscriminate group were subjected to thermal treatment by traditional cooking method of Bangladesh. Oxytetracycline residues had found in cooked meat, liver, and juice part, suggesting that antibiotic residues disseminated to juice part from flesh part after cooking.

**Conclusion:**

Evidence suggests that proper maintenance of withdrawal period would minimize oxytetracycline residues in broiler soft tissues, whereas antibiotics retained in soft tissues of broiler in case of indiscriminate use. Traditional cooking does not change oxytetracycline residual status in edible tissues. Therefore, awareness regarding the proper maintenance of withdrawal period after antibiotic treatment of broiler is one of the best strategies which may positively reduce the risk of antimicrobial drugs residue in meat.

## Introduction

Poultry is one of the most widespread food industries worldwide. This industry is growing largely as a profitable business [1]. Antibiotic drugs are typically used to serve three purposes in poultry; (1) therapeutic use where animals (either individually or in small groups) are administered with high doses of antibiotics for relatively shorter periods, (2) prophylactic use that involves exposure of animals with moderate doses of antimicrobials for longer time durations, and (3) growth promotion where antibiotics in subtherapeutic doses [2,3]. Antibiotics abuse has resulted in drug residues in animal products [4]. Antimicrobial drugs have the potential to leave drug-related residues such as parent drug and their metabolites and/or conjugates in meat, milk, and eggs [5-7]. The antibiotic residues in milk, meat, and egg may persist for longer period after antibiotics treatment, when the administration of antibiotics in low label and extra-label fashion and also not following the specific recommended withdrawal period. The minimum withholding period for milk and egg is 7 days and for meat is as long as 28 days after treated with some antibiotics [8].

Nowadays, oxytetracycline is one of the most widely used antibiotics in poultry sector. Oxytetracycline is widely used for the prevention and control of diseases in poultry industry due to its availability, relatively cheaper price, and more easily use by oral administration through drinking water or feed [9]. It is also used as a growth promoter in animals. The acceptable maximal residual limit (MRLs) for oxytetracycline as recommended by the Joint Food and Agriculture Organization/WHO Expert Committee on Food Additives are 200, 600, and 1200 µg/kg for the liver, muscles, and kidney, respectively [10]. Oxytetracycline is a broad-spectrum antibiotic in the tetracycline class. It was first found near Pfizer Laboratories in a soil sample yielding the soil actinomycetes, *Streptomyces rimosus* by Alexander Finlay during 1950 [11]. About 60% of an ingested dose of oxytetracycline is absorbed from the gastrointestinal tract and widely distributed in the body, particularly to liver, kidney, bones, and teeth [12]. The metabolisms of oxytetracycline are known to bind to plasma proteins at varying degrees in different species of animals [13]. In addition, oxytetracycline has a short half-life (7-10 h) [14].

The presence of antibiotic residue in food above the MRL has been recognized worldwide by various public and government authorities [15]. Antibiotic residue below the MRL level in food products is considered safe for human consumption, but when food products contain antibiotic residue above MRL level or acceptable daily intake level, the food is unsafe for human consumption. When antimicrobial drug residues transmitted to humans through the consumption of contaminated edible tissues, these residues lead to several pathological implications that are considered as major health hazards. A lot of essential antibiotics are employed during poultry production in several countries, threatening the safety of such products (through antimicrobial residues) and the increased possibility of development and spread of microbial resistance in poultry settings [16]. In addition, indiscriminate employment of antimicrobial agents increases the dissemination of resistance against multiple drugs in food-borne bacteria and/or human pathogens, leading to loss of effectiveness of antibiotics against poultry, animals, and human ailments [17]. Since meat is always cooked before consumption, few reports have been published about the effect of cooking or thermal effect on the stability of oxytetracycline residues in meat. The fate of drug residues during heat processing is, however, unclear. Many researchers have been interested in evaluating whether antibiotics residue can be destroyed by cooking procedures, pasteurization, or canning processes [18-20]. In this regard, few studies have been carried out about the thermal effects or effects of traditional cooking on oxytetracycline residual status in cooked meat in Bangladesh.

This study aimed to evaluate whether proper maintenance of withdrawal period does minimize the oxytetracycline residue and whether thermal treatment or traditional cooking has any beneficial effects on antibiotic residual status in meat and liver of antibiotic-treated broiler.

## Materials and Methods

### Ethical approval

The present study design and all experimental procedures were approved and performed according to the guidelines for the care and use of animals as established by Animal Welfare and Experimentation Ethics Committee, Bangladesh Agricultural University, Mymensingh (Approval number: AWEEC/BAU/2018[17-2]).

### Experimental design

A total of 18 day-old chicks (Cobb-500) were bought from the CP Bangladesh Company Ltd., Mymensingh. The birds were vaccinated from the hatchery. The chicks were brooded first at 35°C temperature in the 1^st^ week under intensive care and the temperature was successively decreased to 2.5°C per week. The birds were fed antimicrobial drugs free hand mixed feed and water for the entire experimental period. On the basis of treatment, birds were divided into three different groups on the 20^th^ day: Group A served as the control group (n=6 broiler chicken). Group B served as the discriminate group (n=6 broiler chicken), and broiler chicken was treated with oxytetracycline in their drinking water at the dose rate of 1 g/L daily for 5 consecutive days (20^th^ day-24^th^ day) and 10 days’ withdrawal period was maintained before sacrifice [21]. Group C served as the indiscriminate group (n=6 broiler chicken) and broiler chicken was treated with oxytetracycline in their drinking water at the dose rate of 1 g/L till the sacrifice that is a common practice of administering antibiotics to the broiler close to the time of slaughter.

### Hematological analysis

The blood samples from the broiler of Groups A, B, and C were collected and preserved separately. Total erythrocyte count (TEC), estimation of hemoglobin concentrations (Hb%), and packed cell volume (PCV) estimation were done following the method described by Lamberg and Rothstein [22] and Ripon *et al*. [23].

### Sacrificing and sampling

Broiler chickens were sacrificed on the day of 35 for samples collection. After the slaughtering of the broiler chicken, liver, kidney, spleen, thigh muscle, and breast muscle were collected. The organs were washed with physiological saline to remove debris and clotted blood. Finally, samples were stored in a deep freeze and preserved at −20°C in zip poly bag until further advanced procedures were performed. The polybag containing samples was properly marked with a marker to identify the samples.

### Sample preparation

Four grams of each sample were grinded and blended. The sample was weighted with weight box and taken into a falcon tube. Ten milliliters of phosphate buffer saline (pH 7.2) were added and mixed by vortexing. After that, 2 ml 30% trichloroacetic acid was added and mixed properly by vortex, then centrifuged at 6000 rpm for 20 min. At least 2 ml supernatant was taken. Supernatant was collected and filtered by Whatman filter paper and funnel. Filtrated fluid was collected in another falcon tube and same amount of diethyl ether was added and left for 10 min at room temperature. The bottom layer was collected and pooled carefully into screw cap vial and kept into refrigerator for future analysis as described previously [24,25].

### Standard preparation for selected drug

The standard for oxytetracycline was prepared by dissolving 0.1 g of powder in 4 ml solution of methanol. Standard solution was stored in −4°C as described previously [25].

### Preparation, spotting, and running of thin-layer chromatography (TLC) plate

TLC was performed according to Tajick and Shohreh [26] and also Sattar *et al*. [27] with some adjustments. TLC plate was cut into appropriate size (7 cm×10 cm) from 20 cm×20 cm. A straight line was drawn across the plate approximately 2 cm from the bottom by a pencil. Another straight line was drawn across the plate below 1 cm from the upper edge of the plate. Desired spots marking was marked on the bottom line where analytes were dropped. Spots were applied to the plate using thin capillary glass pipettes. A volume of 10 μl was used for spotting. The plate was placed in TLC tank (contained mobile phase: acetonitrile and methanol; 1:1) and covered by a lid and it was left until the mobile phase reached the upper line. Spots were visualized in UV detection box at 254 nm. Spots marking were done by pencil for the calculation of retention factor (R_f_).

### Calculation of R_f_ values

These measurements are the distance traveled by the solvent, and the distance traveled by individual spots. Same R_f_ value of standard and sample considered similar compound.

### Effect of thermal treatment or traditional cooking

Four grams each of all oxytetracycline residues positive liver and muscle tissues were weighed before traditional cooking or heat treatment. The weighed samples were cooked in a special curry cooker and we cooked our samples in a traditional way of Bangladesh. We used cooking oil with different types of spices such as salt, turmeric, oil, onion, garlic, ginger, and chili for traditional cooking. After cooking, cooked meat portion and juice portion were separately collected for TLC analysis to evaluate the thermal effects on oxytetracycline residual status.

### Preparation of cooked meat samples and TLC analysis

Sample preparation was done both for cooked meat and juice part for screening oxytetracycline residues in both meat and juice part. Sample preparation and TLC analysis were done like raw soft tissue preparation as described before.

### Statistical analysis

Data from the study were analyzed in GraphPad Prism Statistical Software version 8 (GraphPad Software, San Diego, CA., www.graphpad.com). Unpaired t-test was used to compare the mean values of two variables while one-way ANOVA was used to compare the mean values of more than two variables. The alpha value of significance was set at p<0.05.

## Results and Discussion

Poultry sector is an integral part of farming system in Bangladesh. This industry is growing largely as a profitable business. The most widely used poultry for meat is broiler chicken. Therefore, to fulfill the huge demand of protein for large population in Bangladesh, broiler farming is growing rapidly in Bangladesh. Due to the high prevalence of diseases, farmers are using a wide variety of antibiotics in poultry production for therapeutic, prophylactic purposes as well as use as growth promoters indiscriminately without knowing the adverse effects both in public health and environment. Due to indiscriminate use, antibiotic enters into human food chain and causes human health hazards. This research work was, therefore, undertaken to determine whether oxytetracycline residue can be minimized by proper maintenance of withdrawal period of antibiotics or not and to evaluate the antibiotic residual status in meat and liver of antibiotic-treated broiler after thermal treatment or traditional cooking.

### Effects of discriminate and indiscriminate use of oxytetracycline on hematological parameters in broiler chicken

TEC, PCV, and Hb (gm%) analyses were done and highest mean value was found in the control group where no antibiotic was used. The mean values of TEC were 2.51±0.04 for the control group, 2.46±0.02 for the discriminate group, and 2.45±0.02 for the indiscriminate group (Table-1). Hb (gm%) mean values were 7.07±0.10 for the control group, 6.86±0.10 for the discriminate group, and 6.80±0.09 for the indiscriminate group (Table-2). In addition, PCV mean values were 19.83±1.30 for the control group, 18.67±0.92 for the discriminate group, and 17.33±0.42 for the indiscriminate group (Table-3). Analyzed data showed that there is a tend to decrease TEC, PCV, and Hb (%) after discriminate and indiscriminate use of oxytetracycline in broiler. However, there is no significant difference among the group mean values (p>0.05). Trinca *et al*. [28] also showed that there is no significant change of PCV in broilers those receiving overdose of antibiotics. The present study result supports the previous findings of Ognean *et al*. [29] who also observed decreases of TEC. The previous study reported that there is a fall of hematological values (TEC, PCV, and Hb [%]) in baby chicks (1-5 days), whereas in older ages (22-27 days), the values were similar to normal analogs after antibiotics treatment [30]. In agreement with the previous reports stated above, the present study data indicate that discriminate and indiscriminate use of oxytetracycline has no significant influence on hematological parameters in broiler chicken.

**Table-1:** Total erythrocyte count (million/mm^3^) of three individual groups.

Name of group	Total erythrocyte count (million/mm^3^) Mean±SEM	p-value	Level of significance
Control group	2.51±0.04		
Discriminate group	2.46±0.02	0.10	NS
Indiscriminate group	2.45±0.02		

SEM=Standard error mean, NS=Non significant

**Table-2:** Hemoglobin (gm%) of three individual groups.

Name of group	Hemoglobin (gm%) Mean±SEM	p-value	Level of significance
Control group	7.07±0.10		
Discriminate group	6.86±0.10	0.15	NS
Indiscriminate group	6.80±0.09		

SEM=Standard error mean, NS=Non significant

**Table-3:** Packed cell volume (%) of three individual groups.

Name of group	Packed cell volume (%) Mean±SEM	p-value	Level of significance
Control group	19.83±1.30		
Discriminate group	18.67±0.92	0.21	NS
Indiscriminate group	17.33±0.42		

SEM=Standard error mean, NS=Non significant

### Screening of oxytetracycline residue in raw soft tissues after discriminate and indiscriminate use of oxytetracycline

In the present study, broiler chickens of the control group were maintained handmade feed and tap water *ad libitum* and no antibiotic was given to the birds during the experimental periods. Out of 30 samples (liver, kidney, spleen, thigh muscle, and breast muscle from each bird; n=6) of the control group, all samples were negative to the oxytetracycline residue on TLC plate. That means no antibiotic residues were present in soft tissues (Table-4). The discriminate group received oxytetracycline 1 g/L drinking water for 5 consecutive days, and 10 days’ withdrawal period was maintained before sacrifice. No antibiotic residues were found in the discriminate group. However, one sample has found positive from six liver, kidney, and spleen samples, these positive results may be due to some technical error or may be due to individual birds’ physiology. Here, TLC results (Table-5) revealed that out of 30 samples, only three samples showed positive results and total positive percentage was 10%. The other 27 samples showed negative results to the oxytetracycline residue and the negative percentage was 90%. These results suggested that no antibiotic residues were found if antibiotics are being used with strictly maintained withdrawal period in broiler farm. The indiscriminate group received oxytetracycline 1 g/L drinking water till their sacrificed day, where withdrawal period was not maintained. All the samples from the indiscriminate group showed positive for oxytetracycline residues. Here, Table-6 revealed that 100% of samples were positive to oxytetracycline residues. The present study results are in agreement with Razia *et al*. [31], who reported that declared withdrawal period of oxytetracycline (7 days) and safe level of oxytetracycline are almost nearest position. They have found oxytetracycline residues in edible tissues if proper withdrawal period is not maintained before sacrifice. In addition, the previous study also shown that if antibiotic is used in an indiscriminate way, a common practice of administering antibiotics to the broiler close to the time of slaughter, antibiotic residue can be found in tissues and this is most concerning [32]. In this regard, Kabir *et al*. [32] also reported that the deposition of antibiotic residues in edible tissues of broiler are significantly higher when withdrawal period is not maintained properly.

**Table-4:** Oxytetracycline residue in different samples of control group.

Sample	Total number of samples	Number of samples		% of sample	
	
		Positive	Negative	Positive (%)	Negative (%)
Liver	6	0	6	0.00	100.00
Kidney	6	0	6	0.00	100.00
Spleen	6	0	6	0.00	100.00
Thigh muscle	6	0	6	0.00	100.00
Breast muscle	6	0	6	0.00	100.00
Total	30	0	30	0.00	100.00

**Table-5:** Oxytetracycline residues in different soft tissues of discriminate antibiotic group.

Sample	Total number of samples	Number of samples		% of sample	
	
		Positive	Negative	Positive (%)	Negative (%)
Liver	6	1	5	16.67	83.33
Kidney	6	1	5	16.67	83.33
Spleen	6	1	5	16.67	83.33
Thigh muscle	6	0	6	0.00	100.00
Breast muscle	6	0	6	0.00	100.00
Total	30	3	27	10.00	90.00

**Table-6:** Oxytetracycline residues in different soft tissues of indiscriminate antibiotic group.

Sample	Total number of samples	Number of samples		% of sample	
	
		Positive	Negative	Positive (%)	Negative (%)
Liver	6	6	0	100.00	0.00
Kidney	6	6	0	100.00	0.00
Spleen	6	6	0	100.00	0.00
Thigh muscle	6	6	0	100.00	0.00
Breast muscle	6	6	0	100.00	0.00
Total	30	30	0	100.00	0.00

### Thermal effect on oxytetracycline residual status in edible tissues

**Figure-1:**
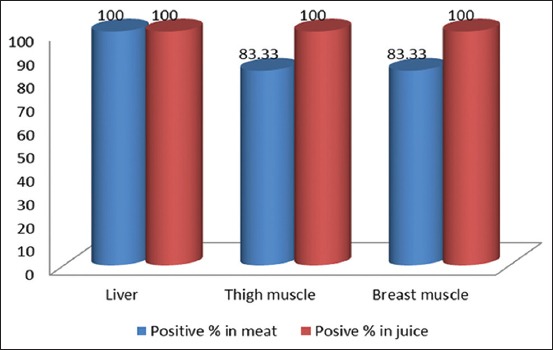
Positive percentage of oxytetracycline residue in different cooked meat samples and their curry juice parts.

After the screening of raw liver, kidney, spleen, and muscles of six broiler chickens from the indiscriminate group, six liver samples, six kidney samples, six spleen samples, six thigh muscle samples, and six breast muscle samples were found positive to oxytetracycline residues on TLC plate. Among all oxytetracycline residue positive samples, six liver samples, six thigh muscle samples, and six breast muscle samples were used to evaluate the thermal effect on oxytetracycline residues. For this purpose, positive liver and muscle samples were cooked by traditional cooking methods. After cooking, the samples were cooled at room temperature and both flesh and curry juice were separately analyzed for oxytetracycline residue by TLC technique. Here, out of total 18 cooked samples, 6 liver, 5 breast muscle, and 5 thigh muscle samples with their juice part were found positive to oxytetracycline residue in both cooked meat part and curry juice part (Figure-1). These results suggested that after cooking or heat treatment, oxytetracycline residues disseminated into curry juice from flesh part. When cooked by traditional cooking method, out of six thigh muscles and breast muscle samples, all their juice parts were found positive; however, 1 (16.67%) thigh muscle and breast muscle samples were negative to oxytetracycline residue (Figure-1). These results may be due to dissemination of oxytetracycline residue from flesh part to juice part. The present study results are in consistent with the result of Vivienne *et al*. [33], they also found that cooking is more effective in reducing the concentration of oxytetracycline residue in meat and a great percentage of residue disseminate to juice part. In agreement with above previously reported data, the present study showed that antibiotic residues retained in meat and juice part even after cooking, suggesting that the exposure to residues may be minimized by discarding curry juice part after cooking. Hussein and Khalil [34] monitored the effect of cooking process on antibiotic residue levels in broiler fillet and showed that cooking had an effect in reducing the concentration of antibiotic residues as there is a significant reduction in the percentages of oxytetracycline in cooked meat. These results suggested that heat treatment by cooking does not destroy the residual status in edible tissues and their metabolites or conjugates may have remained in cooked meat and their curry juice part after boiling. The present study result indicates that oxytetracycline does not destroy by heat treatment which merged with published literature results [35-37]. In this regard, the previous report showed that heat-labile antibiotic residues can be destroyed by cooking procedures, pasteurization, or canning processes; however, antibiotics metabolites and/or conjugates may have remained in milk after boiling [5-7]. Therefore, further molecular detail studies are needed to find out whether antibiotic residues in cooked meat and their curry juice part are in active form or its metabolites or its conjugates.

The limitation of our study was small sample size. In addition, the concentration of oxytetracycline antibiotic residue in tissues of broiler chicken as well as in cooked meat with their curry juice parts was not quantified. Therefore, we did not compare oxytetracycline residues present in tissues with the maximal residual limit (MRL) after cooking. Finally, further research is needed to address the issue of whether cooking may beneficial effects reducing antibiotic residues in edible tissues.

## Conclusion

The present study showed that there is no oxytetracycline residue in broiler edible tissues when proper withdrawal period is maintained after the use of oxytetracycline. In contrast, there is oxytetracycline residue in broiler liver, kidney, spleen, and muscles during indiscriminate use of oxytetracycline that means the administration of antibiotics to the broiler close to the time of slaughter. This result indicates that the use of antibiotic with a proper guideline or with proper maintaining withdrawal period can minimize antibiotic residues in meat. Discriminate and indiscriminate use of oxytetracycline do not have any significant impact on hematological parameters. In addition, oxytetracycline residue was still present after traditional cooking in broiler meat as well as curry juices, indicating that traditional cooking does not change oxytetracycline residual status in edible tissues. Therefore, in this regard, community-based awareness building programs, training, proper monitoring, and legal steps by the government authorities can play an important role to reduce antibiotic residues in broiler meat for food safety.

## Authors’ Contributions

MRAF and MRA performed the experiments and wrote the manuscript; KR and MZI designed and supervised the research work; KR revised and finalized the draft of manuscript; SHK, MAM, TTA, and MTH were responsible for data analysis, graph, and table preparation and did the statistical analysis and revised the draft. All authors read and approved the final version.
